# Effects of Heat Stress on Production Performance, Redox Status, Intestinal Morphology and Barrier-Related Gene Expression, Cecal Microbiome, and Metabolome in Indigenous Broiler Chickens

**DOI:** 10.3389/fphys.2022.890520

**Published:** 2022-04-29

**Authors:** Wen-Chao Liu, Zi-Yi Pan, Yue Zhao, Yan Guo, Sheng-Jian Qiu, Balamuralikrishnan Balasubramanian, Rajesh Jha

**Affiliations:** ^1^ Department of Animal Science, College of Coastal Agricultural Sciences, Guangdong Ocean University, Zhanjiang, China; ^2^ Department of Food Science and Biotechnology, College of Life Science, Sejong University, Seoul, South Korea; ^3^ Department of Human Nutrition, Food and Animal Sciences, College of Tropical Agriculture and Human Resources, University of Hawaii at Manoa, Honolulu, HI, United States

**Keywords:** antioxidant capacity, cecal microbiota, cecal metabolome, heat stress, intestinal barrier function, slow-growing broilers

## Abstract

This study was done to evaluate the effects of heat stress (HS) on production performance, redox status, small intestinal barrier-related parameters, cecal microbiota, and metabolome of indigenous broilers. A total of forty female indigenous broilers (56-day-old) were randomly and equally divided into normal treatment group (NT group, 21.3 ± 1.2°C, 24 h/day) and HS group (32.5 ± 1.4°C, 8 h/day) with five replicates of each for 4 weeks feeding trial. The results showed that the body weight gain (BWG) of broilers in HS group was lower than those in NT group during 3–4 weeks and 1–4 weeks (*p* < 0.05). The HS exposure increased the abdominal fat rate (*p* < 0.05) but decreased the thigh muscle rate (*p* < 0.01). Besides, broilers in HS group had higher drip loss of breast muscle than NT group (*p* < 0.01). Broilers exposed to HS had lower total antioxidant capacity (T-AOC) in serum and jejunum, activities of total superoxide dismutase (T-SOD) in the jejunum, glutathione peroxidase (GSH-Px) in the thigh muscle, duodenum, and jejunum; and catalase (CAT) in breast muscle, duodenum, and jejunum (*p* < 0.05). Whereas the malondialdehyde (MDA) contents in breast muscle, duodenum, and jejunum was elevated by HS exposure (*p* < 0.05). Moreover, the relative mRNA expression of *Occludin* and *ZO-1* in the duodenum, *Occludin, Claudin-1, Claudin-4, ZO-1, Mucin-2* in the jejunum, and the *Claudin-4* and *Mucin-2* in the ileum was down-regulated by HS exposure (*p* < 0.05). The 16S rRNA sequencing results showed that the HS group increased the relative abundance of *Anaerovorax* in the cecum at the genus level (*p* < 0.05). Cecal metabolomics analysis indicated 19 differential metabolites between the two groups (*p* < 0.10, VIP >1). The Kyoto Encyclopedia of Genes and Genomes (KEGG) analysis revealed that the differential metabolites mainly enriched in 10 signaling pathways such as the Citrate cycle (TCA cycle) (*p* < 0.01). In summary, chronic HS exposure caused a decline of production performance, reduced antioxidant capacity, disrupted intestinal barrier function, and negatively affected cecal microbiota and metabolome in indigenous broilers.

## Introduction

With global warming, the ambient temperature has gradually risen worldwide in the past decades. The high temperature has become an environmental hazard with a wide range of detrimental effects on poultry production (Vandana et al., 2021). Heat stress (HS) is the primary harmful consequence caused by high-temperature climates. Because of their strong metabolism, being covered with feathers, and being unable to sweat, broiler chickens are susceptible to HS (Wasti et al., 2020; Goel, 2021). It is well known that HS exposure leads to a reduction in growth performance, which is associated with the loss of appetite and decreased feed intake, also can be ascribed to the deviation of energy resources from production to adaptation pathway in broilers (Gogoi et al., 2021). Besides, under HS situations, systemic disorders of physiology and metabolism appear in broilers, such as the body temperature rises, peripheral blood flow increases, and lipid peroxidation aggravates (Guo et al., 2021a; Guo et al., 2021b; Chauhan et al., 2021). These unfavorable changes in physiological functions have a significant influence on production performances, including but not limited to growth rate, carcass traits, meat yield, and quality (Liu et al., 2019; Emami et al., 2021; Ma et al., 2021), and a detrimental effect on organ health of broilers (Lara and Rostagno, 2013; Liu et al., 2021a).

The gastrointestinal tract has dual functions, which is not only a place for nutrients digestion and absorption but also an innate barrier to maintain homeostasis (Jha et al., 2019; Liu W.-C. et al., 2020). Gut health is particularly vulnerable to HS, which are in line with previous studies showing that HS-induced impairment of intestinal morphology (Song et al., 2013), damage to tight junction structure (Zhang et al., 2017), and imbalance of gut mucosal redox status (Liu G. et al., 2020). These alterations boost intestinal permeability and permit the translocation of pathogens and toxins present in the gut lumen, ultimately leading to intestinal dysfunction (Rostagno, 2020). On the other hand, there is a complex community of microbiota in the intestine, and gut microbiota plays a significant role in nutrient digestion and barrier regulation (Yadav and Jha, 2019). The cecum is a part of the distal intestine and rich in microorganisms among the gut segments, which is also the site for indigestible fiber fermentation (Yadav et al., 2021). The fermentation products produced by cecal microbes positively affect intestinal health, and the numerous cecal metabolites play pivotal roles in maintaining the intestinal barrier function (Jha and Mishra, 2021; Singh et al., 2021). It has been demonstrated that broilers subjected to HS had negative influences on the cecal microbial community and metabolites, but the changes were partially inconsistent (Wang et al., 2018; Liu G. et al., 2020; Wang G. et al., 2021; Wasti et al., 2021a; Wasti et al., 2021b), indicating that further investigations are required.

Indigenous yellow-feathered broilers are slow-growing breeds but have excellent meat quality, and the meat of this type of broilers has become more popular among Chinese consumers in recent years (Wang et al., 2019). Therefore, the production scale of yellow-feathered broilers continues to expand in China. At present, to satisfy the consumer preferences regarding the flavor, the annual production of yellow-feathered broilers is approximately four billion, almost the same as that of fast-growing broilers in China (Wang Y. et al., 2021). Interestingly, indigenous chickens exhibit better heat tolerance than fast-growing broilers, such as Ross/Cobb broilers, due to their slower growth and lower metabolic rate (Xu et al., 2018). However, existing studies mainly focused on fast-growing commercial broilers, and only a few researches revealed that HS has detrimental effects on metabolic status and gut health in Chinese indigenous broilers (Guo et al., 2021a; Liu et al., 2021b). Furthermore, in indigenous broilers, the effect of HS on detailed pathobiology, especially the gut microbiome and metabolome, is not studied well. Therefore, the current study aimed to explore the effects of HS on production performance, redox status, small intestinal barrier parameters, cecal microbiota, and metabolome in native Chinese broiler chickens.

## Materials and Methods

### Birds, Experimental Design, and Management

A total of forty female Huaixiang chickens (Chinese indigenous broiler breed, slow-growing, and yellow-feathered type) at 8 weeks old were obtained from local farms in Zhanjiang, Guangdong, and used in this study. The age selection of broilers was made considering that indigenous broilers enter the growing-finishing stage after 8-weeks of age, and growing-finishing broilers are more sensitive to HS for production (Wasti et al., 2020). The female broilers were only used to exclude the influence of gender on the experiment. The birds with initial average body weight (BW) of 840.75 ± 20.79 g were randomly and equally divided into two treatments in a completely randomized design. The treatments included normal treatment group (NT) (21.3 ± 1.2°C throughout the experimental period, thermoneutral zone) and HS group (32.5 ± 1.4°C, 8 h/day, from 9:00 a.m. to 17:00 p.m.). Each group had five replications with four broilers per replicate and was studied for 4 weeks. The relative humidity of NT and HS were maintained at 55–70%. The temperature of the two groups was controlled by environmental control equipment, including a dehumidifier (SHIMEI, MS-9138BE, Guangzhou, China), humidifier (OROSIN, DRST-03AE, Guangzhou, China), air conditioner (GREE, KF-120LW, Zhuhai, China), and heater (MIDEA, NPS7-15A5, Zhongshan, China). The chickens were kept in three-layer wire-cages of 90 (length) × 70 (width) × 40 (height) cm and were ensured that all chickens had free access to water and feed. The plastic trays were used under each cage to collect the excrements, and the excrements were manually cleaned twice a day. All broilers were fed a corn-soybean meal basal diet to meet the nutrient requirements recommended by the Chinese chicken breeding standard (NY/T33-2004). The composition and nutrient contents of diet were as reported in our previous study (Guo et al., 2021a).

### Growth Performance and Carcass Traits Determination

The feed intake was recorded regularly. Briefly, the amount of feed supplied and leftover were recorded daily, and the data were used to calculate daily feed intake. Meanwhile, the broilers were weighted in a cage at the start and the end of weeks 2 and 4. The daily feed intake and body weight data were used to calculate the body weight gain (BWG), feed intake, and feed/gain for 1–2, 3–4, and 1–4 weeks of the study period. At the end of week 4, one chicken from each replicate was selected randomly and slaughtered by neck bleeding. Subsequently, the partial carcass traits, including the abdominal fat (%), breast muscle (%), and thigh muscle (%), were determined. The carcass traits were determined as previously reported (Liu et al., 2019).

### Measurement of Meat Quality

After the selected broiler from each replicate cage was slaughtered, the left side of breast muscle (pectoralis major) and the left side of thigh muscle (biceps femoris) were collected for the determination of meat quality, including cooking loss (%), drip loss (%), and pH at 45 min and 24 h after slaughter. The meat quality determination was done as in previous studies (Berri et al., 2008; Liu et al., 2019).

### Antioxidant Capacity Analysis

To analyze the antioxidant capacity of broilers, 10 g of liver samples, breast and thigh muscle samples from the right side, and mucosal samples of duodenum, jejunum, and ileum were collected from one bird per replicate; 5 ml blood was collected from the wing vein using a vacuum tube without anticoagulant (one chicken per replicate), and the serum samples were obtained by centrifugation at 4,000 rpm (2 min, 4 °C) after blood coagulation. Afterward, the total anti-oxidation capacity (T-AOC), malondialdehyde (MDA) contents, and antioxidant enzymes activity, including the total superoxide dismutase (T-SOD), glutathione peroxidase (GSH-Px), and catalase (CAT), were analyzed using commercial kits (Nanjing Jiancheng Bioengineering Institute, Nanjing, China) following the manufacturer’s instructions. The catalog numbers of the kits are as follows: T-AOC, A015-2-1; MDA, A003-1-2; T-SOD, A001-3-2; GSH-Px, A005-1-2; CAT, A007-1-1.

### Determination of Intestinal Morphology and Barrier Function Related Genes Expression

For intestinal morphology analysis, approximately 2 cm segments located in the middle of the duodenum, jejunum, and ileum (one broiler from each replicate) were collected, fixed in 4% paraformaldehyde for 48 h. The intestinal segments were mounted with paraffin sections and stained with hematoxylin and eosin (H & E). Subsequently, the sections were observed using an inverted optical microscope (SDPTOP, GD-30RFL, Guangzhou, China) under ×40 magnification. The intestinal morphology parameters, villus height, villus width, and crypt depth were measured using T-Capture Imaging Application 4.3 software, and the villus height/crypt depth ratio was calculated accordingly. The villus surface area was calculated following the formula (2π) × (villus width/2) × villus height (de los et al., 2005).

Total RNA was extracted from the mucosal samples of the duodenum, jejunum, and ileum using RNA extraction kits (catalog No. N066, Jiancheng Bioengineering Institute, Nanjing, China). The reverse transcription of cDNA from RNA using RT reagent kits (catalog No. RR047A, TaKaRa Biotechnology Co., Ltd, Shiga, Japan). Then the quantitative real-time PCR (qPCR) was performed to detect the mRNA expression of intestinal barrier function-related genes, including *E-cadherin, Occludin, Claudin-1, Claudin-4, ZO-1,* and *Mucin-2*. The CFX-96 real-time PCR detection system (BioRad, Irvine, CA, United States) was used for the qPCR reaction. The reaction system and conditions were as in our previous study (Liu et al., 2021c). β-actin was used as an internal reference gene; the primer’s information is presented in Table 1. Finally, the relative mRNA expression levels of the intestinal barrier function-related genes were calculated using the 2^−ΔΔCt^ method (Livak and Schmittgen, 2001).

**TABLE 1:** Nucleotide sequences of the specific primers used in real-time qPCR analyses.

Genes	Accession No	Sequence
*E-cadherin*	NM 001039258.2	F: CGA​CAA​CAT​TCC​CAT​CTT​CA
		R: CACCATCCAGGTTCCCAC
*Occludin*	NM 205128.1	F: CTG​CTG​TCT​GTG​GGT​TCC​T
		R: CCA​GTA​GAT​GTT​GGC​TTT​GC
*Claudin-1*	NM 001013611.2	F: ATG​ACC​AGG​TGA​AGA​AGA​TGC
		R: TGCCCAGCCAATGAAGAG
*Claudin-4*	AY435420.1	F: AGG​ACG​AGA​CAG​CCA​AAG​C
		R: CACGTAGAGCGACGAGCC
*ZO-1*	XM 413773.4	F: CGT​AGT​TCT​GGC​ATT​ATT​CGT
		R: TGGGCACAGCCTCATTCT
*Mucin-2*	XM 421035	F: TGA​GTC​AGG​CAT​AAA​TCG​TGT
		R: CAG​GTC​TAA​GTC​GGG​AAG​TGT​A
*β-actin*	NM 205518.1	F: TTG​GTT​TGT​CAA​GCA​AGC​GG
		R: CCC​CCA​CAT​ACT​GGC​ACT​TT

### Cecal Microbial Community Analysis

After slaughter, approximately 5 g of cecal digesta samples were collected from one chicken per replicate. The microbial DNA of cecal digesta was extracted using QIAamp DNA Stool Mini Kit (catalog No. 51306, QIAGEN, CA, Hamburg, Germany). The V3-V4 regions of the 16S rRNA genes were sequenced on the Illumina MiSeq platform by Personalbio Technology Co., Ltd. (Shanghai, China). The primer pairs were 338F (5′-ACT​CCT​ACG​GGA​GGC​ACA​G-3′) and 806R (5′- GGACTACHVGGGTWTCTAAT-3′). The raw reads were demultiplexed and quality-filtered using FLASH software (v1.2.7, http://ccb.jhu.edu/software/FLASH/) and QIIME software (v1.8.0 Quantitative Insights Into Microbial Ecology, http://qiime.org/). Then the obtained effective reads were assigned to operational taxonomic units (OTUs) with 97% similarity using UCLUST sequence alignment tool of QIIME software (v1.8.0). The bacterial taxonomic information corresponding to each OTU was obtained from the Greengenes database (Release 13.8, http://greengenes.secondgenome.com/). The diversity, composition, and difference of the cecal microbial community were analyzed using the Gene Cloud Analysis Platform (QIIME 1, https://www.genescloud.cn) of Personalbio Technology Co., Ltd. (Shanghai, China).

### Metabolomics Analysis in Cecal Digesta

The 1 g of cecal digesta was used for untargeted metabolomics analysis using liquid chromatography-mass spectrometry/mass spectrometry (LC-MS/MS) method and detected by LC-MS/MS system (Personalbio Technology Co., Ltd. Shanghai, China). The experimental procedure included metabolites extraction, LC-MS/MS detection, and data analysis. The metabolites of cecal digesta were extracted using methanol/acetonitrile/water solution (4:4:2, v/v). Then, 2 μl of the supernatant from each sample (one chicken in each replicate) was separated on an Agilent 1290 Infinity LC Ultra High-Performance Liquid Chromatography System (UHPLC) with HILIC column (Agilent Technologies Inc. CA, United States). The conditions of UHPLC were as follows: column temperature 25°C; flow rate 0.3 ml/min; injection volume 2 μl; mobile phase composition A: water +25 mM ammonium acetate +25 mM ammonia water, B: acetonitrile; gradient elution procedure is as follows: 0–1 min, 95% B; 1–14 min, B linearly changes from 95 to 65%; 14–16 min, B linearly changes from 65 to 40%; 16–18 min, B maintained at 40%; 18–18.1 min, B linearly changed from 40 to 95%; 18.1–23 min, B maintained at 95%. The samples were separated by UHPLC and then analyzed by mass spectrometry using a Triple TOF 6600 mass spectrometer (AB SCIEX Inc. MA, United States). The conditions of MS analysis were as follows: Ion Source Gas1 (Gas1): 60, Ion Source Gas2 (Gas2): 60, Curtain gas (CUR): 30, source temperature: 600°C, IonSapary Voltage Floating (ISVF) ±5500 V (plus and minus two mode); TOF MS scan m/z range: 60–1000 Da, Production scan m/z range: 25–1000 Da, TOF MS scan accumulation time 0.20 s/sPectra, Production scan accumulation time 0.05 s/sPectra; Secondary mass spectrometry was acquired using information dependent acquisition (IDA) and used high sensitivity mode, Declustering potential (DP): ±60 V (both positive and negative modes), Collision Energy: 35 ± 15 eV, IDA settings are as follows Exclude isotoPes within 4 Da, Candidate ions to monitor Per cycle: 6. After LC-MS/MS detection, The raw data were converted into mzXML format by ProteoWizard, and the XCMS program was used for peak alignment, retention time correction and extraction of peak areas. The structure identification of metabolites uses accurate mass matching (<25 ppm) and secondary spectrum matching to search the laboratory’s self-built database. For data extracted by XCMS, ion peaks with >50% missing values within the group were removed. The software SIMCA-P 14.1 (Umetrics, Umea, Sweden) was used for pattern recognition. After the data was preprocessed by Pareto-scaling, multi-dimensional statistical analysis was performed, including unsupervised principal component analysis (PCA) analysis, and supervised partial least squares discriminant analysis (PLS-DA) and Orthogonal Partial Least Squares Discriminant Analysis (OPLS-DA). Statistical analysis included Student’s t-test and fold variation analysis. The OPLS-DA model variable importance projection (VIP) > 1 and *p* < 0.10 were used as the criteria to screen significant differential metabolites, and subsequently, cluster analysis and Kyoto Encyclopedia of Genes and Genomes (KEGG) metabolic pathway analysis were performed on the differential metabolites.

### Statistical Analysis

All the statistical analysis of data was by done using SAS 9.4 (SAS, 2013. SAS Institute Inc. Cary, NC, United States). Student’s t-test was performed for comparing the differences between the two groups. Data results were expressed as mean ± standard error. The probability (*P*) value less than 0.05 was considered to be significant, and the *p* value between 0.05–0.10 was considered to be a trend of significance.

## Results

### Growth Performance and Carcass Traits

The results of growth performance are presented in Table 2. During 1–2 weeks, HS exposure tended to reduce the BWG (*p* = 0.070). During 3–4 weeks, HS exposure decreased the BWG (*p* < 0.05) and tended to reduce the feed intake (*p* = 0.084). During the overall study period (1–4 weeks), HS exposure led to a reduction in BWG (*p* < 0.05) and tended to decrease the feed intake (*p* = 0.078) but increased the feed/gain (*p* = 0.057). As shown in Table 2, broilers exposed to HS for 4 weeks had a higher abdominal fat compared with NT group (*p* < 0.001), and the broilers under 4 weeks of HS resulted in a lower thigh muscle (*p* < 0.01).

**TABLE 2:** Effects of heat stress on growth performance and carcass traits in indigenous broilers.

Items	NT Group	HS Group	*p*-Value
Growth performance			
1–2 weeks			
BWG, g	293.00 ± 12.91	255.49 ± 14.56	0.070
Feed intake, g	1341.24 ± 27.19	1243.60 ± 53.51	0.124
Feed/gain	4.64 ± 0.18	4.91 ± 0.10	0.210
3–4 weeks			
BWG, g	292.50 ± 10.09	259.48 ± 10.86	0.039
Feed intake, g	1445.73 ± 30.50	1350.35 ± 42.23	0.084
Feed/gain	4.99 ± 0.17	5.24 ± 0.13	0.250
Overall (1–4 weeks)			
BWG, g	585.45 ± 14.31	514.98 ± 22.44	0.016
Feed intake, g	2,786.77 ± 46.63	2,594.00 ± 92.03	0.078
Feed/gain	4.78 ± 0.12	5.06 ± 0.07	0.057
Carcass traits, %			
Abdominal fat	1.22 ± 0.15	2.89 ± 0.22	<0.001
Breast muscle	9.45 ± 0.62	8.52 ± 0.63	0.322
Thigh muscle	12.47 ± 0.51	8.37 ± 0.74	0.002

NT, normal treatment group, the broilers reared at 21.3 ± 1.2°C throughout the experimental period; HS, the broilers reared at 32.5 ± 1.4 °C for 8 h/day (9:00 a.m. to 17:00 p.m.); BWG, body weight gain. There were 5 replicate cages (20 individuals) per group for determination of growth performance and 5 selected broilers per group for the determination of carcass traits.

### Meat Quality

The meat quality parameters of breast and thigh muscle were presented in Table 3. Broilers in HS group had a higher drip loss after 1, 2, 3 days (*p* < 0.01) and lower pH after 45 min (*p* < 0.05) of breast muscle than those in NT group. Compared with NT group, HS exposure caused an increasing trend in drip loss after 1 day (*p* = 0.076) and reduced the pH after 45 min (*p* < 0.05) of the thigh muscle.

**TABLE 3:** Effects of heat stress on meat quality in indigenous broilers.

Items	NT Group	HS Group	*p*-Value
Breast muscle			
Cooking loss, %	10.56 ± 1.07	13.79 ± 3.28	0.376
Drip loss, %			
1 day	0.90 ± 0.18	3.74 ± 0.43	<0.001
2 days	1.96 ± 0.30	4.81 ± 0.47	<0.001
3 days	2.98 ± 0.31	5.53 ± 0.51	0.003
pH			
45min	6.84 ± 0.01	6.68 ± 0.06	0.035
24h	6.62 ± 0.09	6.49 ± 0.15	0.475
Thigh muscle			
Cooking loss, %	20.04 ± 4.80	24.61 ± 3.65	0.471
Drip loss, %			
1 day	0.59 ± 0.17	2.17 ± 0.75	0.076
2 days	1.44 ± 0.19	2.33 ± 0.86	0.342
3 days	1.99 ± 0.23	2.93 ± 0.90	0.344
pH			
45min	6.97 ± 0.05	6.69 ± 0.09	0.025
24h	6.90 ± 0.08	6.84 ± 0.07	0.572

NT, normal treatment group, the broilers reared at 21.3 ± 1.2 °C throughout the experimental period; HS, the broilers reared at 32.5 ± 1.4 °C for 8 h/day (9:00 a.m. to 17:00 p.m.). There were 5 selected broilers per group for the determination of meat quality.

### Antioxidant Capacity

**FIGURE 1:**
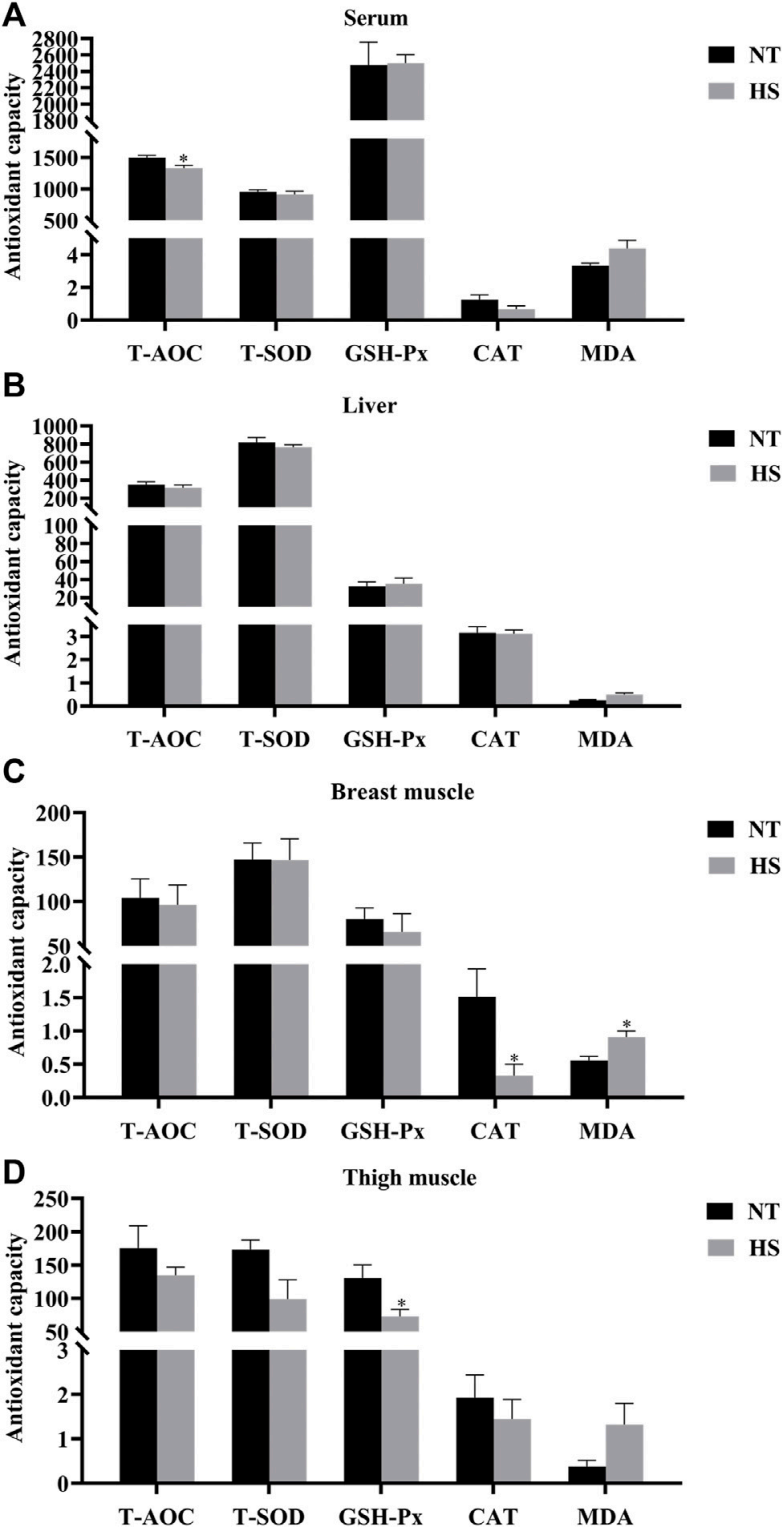
Effects of heat stress on antioxidant capacity of serum **(A)**, liver **(B)**, breast muscle **(C)**, and thigh muscle **(D)** in indigenous broilers (n = 5/group). T-AOC, total antioxidant capacity. T-SOD, total superoxide dismutase. GSH-Px, glutathione peroxidase. CAT, catalase. MDA, malondialdehyde. NT, normal treatment group (21.3 ± 1.2°C, 24 h/day), HS, heat stress group (32.5 ± 1.4°C, 8 h/day). *p < 0.05.

**FIGURE 2:**
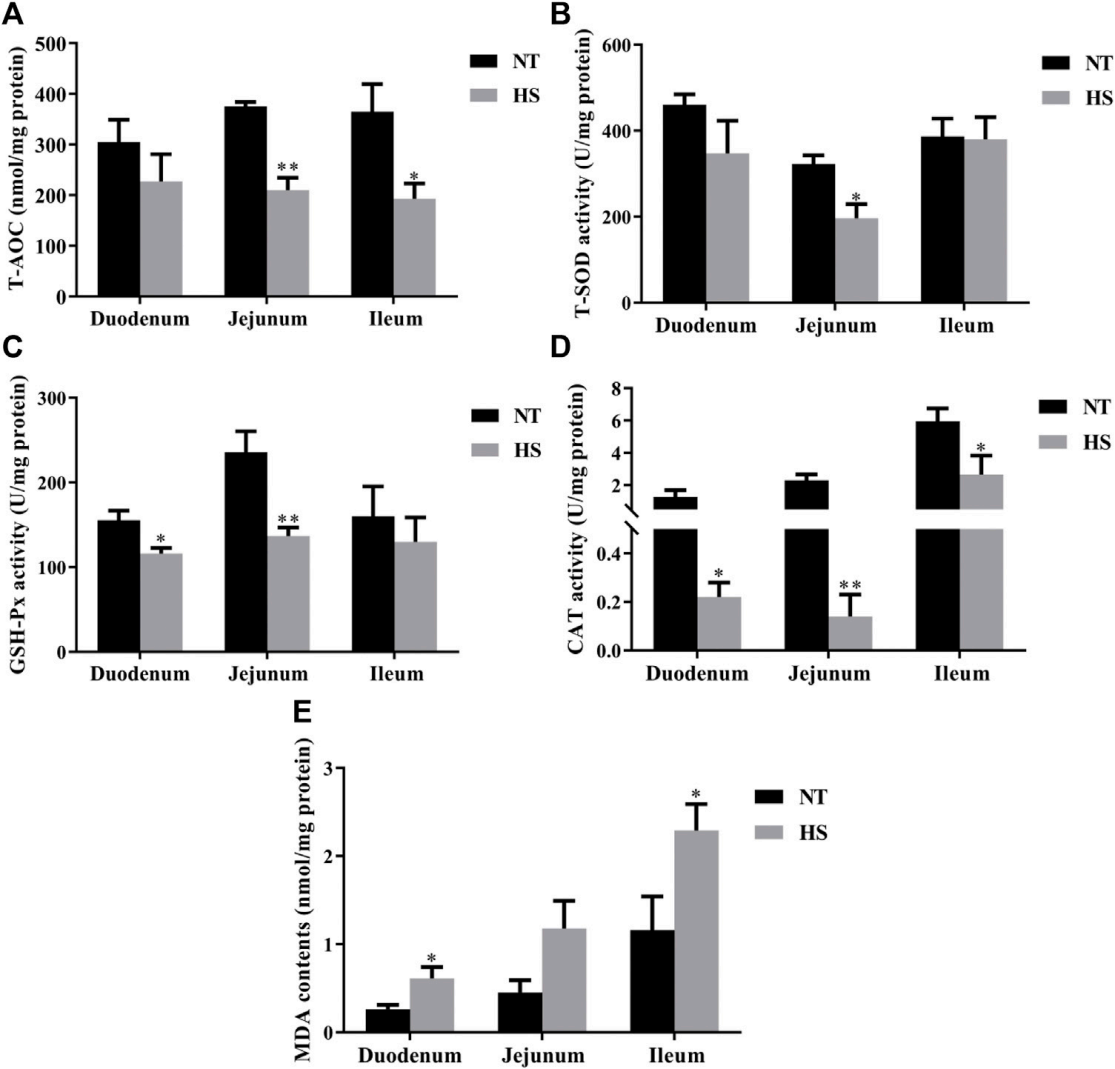
Effects of heat stress on antioxidant capacity of duodenum, jejunum, and ileum in indigenous broilers (n = 5/group). T-AOC, total antioxidant capacity **(A)**. T-SOD, total superoxide dismutase **(B)**. GSH-Px, glutathione peroxidase **(C)**. CAT, catalase **(D)**. MDA, malondialdehyde **(E)**. NT, normal treatment group (21.3 ± 1.2°C, 24 h/day), HS, heat stress group (32.5 ± 1.4°C, 8 h/day). *p < 0.05, **p < 0.01.

As illustrated in Figure 1, broilers in HS group had lower T-AOC in serum, CAT activity in breast muscle, and GSH-Px in thigh muscle than those in NT group (*p* < 0.05). However, the MDA contents of breast muscle were increased by HS exposure (*p* < 0.05). Besides, the HS exposure reduced the mucosal T-AOC in jejunum (Figure 2, *p* < 0.01) and ileum (*p* < 0.05), decreased the mucosal T-SOD activity in jejunum (*p* < 0.05), GSH-Px activity in duodenum (*p* < 0.05) and jejunum (*p* < 0.01), and CAT activity in duodenum (*p* < 0.05), jejunum (*p* < 0.01) and ileum (*p* < 0.05). Compared with NT group, broilers under HS had higher mucosal MDA levels in the duodenum and ileum (*p* < 0.05).

### Intestinal Morphology and Barrier Function Related Genes Expression

The results of intestinal morphology are presented in Table 4. Broilers in the HS group had a lower villus height of duodenum and ileum than NT group (*p* < 0.05). Additionally, the decreased villus height/crypt depth ratio in the ileum was found in broilers under HS (*p* < 0.05).

**TABLE 4:** Effects of heat stress on intestinal morphology in indigenous broilers.

Items	NT Group	HS Group	*p*-Value
Duodenum			
Villus height, μm	1806.82 ± 26.85	1632.74 ± 43.80	0.010
Crypt depth, μm	382.77 ± 46.46	391.69 ± 38.60	0.886
Villus height/crypt depth ratio	5.02 ± 0.61	4.35 ± 0.49	0.422
Villus width, μm	313.15 ± 24.46	279.92 ± 22.63	0.348
Villus surface area, mm^2^	1.78 ± 0.14	1.44 ± 0.15	0.143
Jejunum			
Villus height, μm	1289.70 ± 86.92	1163.22 ± 62.97	0.273
Crypt depth, μm	407.81 ± 35.47	354.63 ± 29.89	0.285
Villus height/crypt depth ratio	3.22 ± 0.25	3.34 ± 0.23	0.733
Villus width, μm	300.45 ± 22.43	294.68 ± 26.99	0.873
Villus surface area, mm^2^	1.21 ± 0.10	1.08 ± 0.12	0.447
Ileum			
Villus height, μm	900.16 ± 21.28	716.79 ± 73.55	0.044
Crypt depth, μm	206.75 ± 15.93	207.13 ± 10.91	0.985
Villus height/crypt depth ratio	4.44 ± 0.28	3.46 ± 0.29	0.042
Villus width, μm	335.18 ± 47.32	319.28 ± 58.22	0.838
Villus surface area, mm^2^	0.94 ± 0.12	0.68 ± 0.09	0.109

NT, normal treatment group, the broilers reared at 21.3 ± 1.2 °C throughout the experimental period; HS, the broilers reared at 32.5 ± 1.4°C for 8 h/day (9:00 a.m. to 17:00 p.m.). There were 5 selected broilers per group for the determination of intestinal morphology.

**FIGURE 3:**
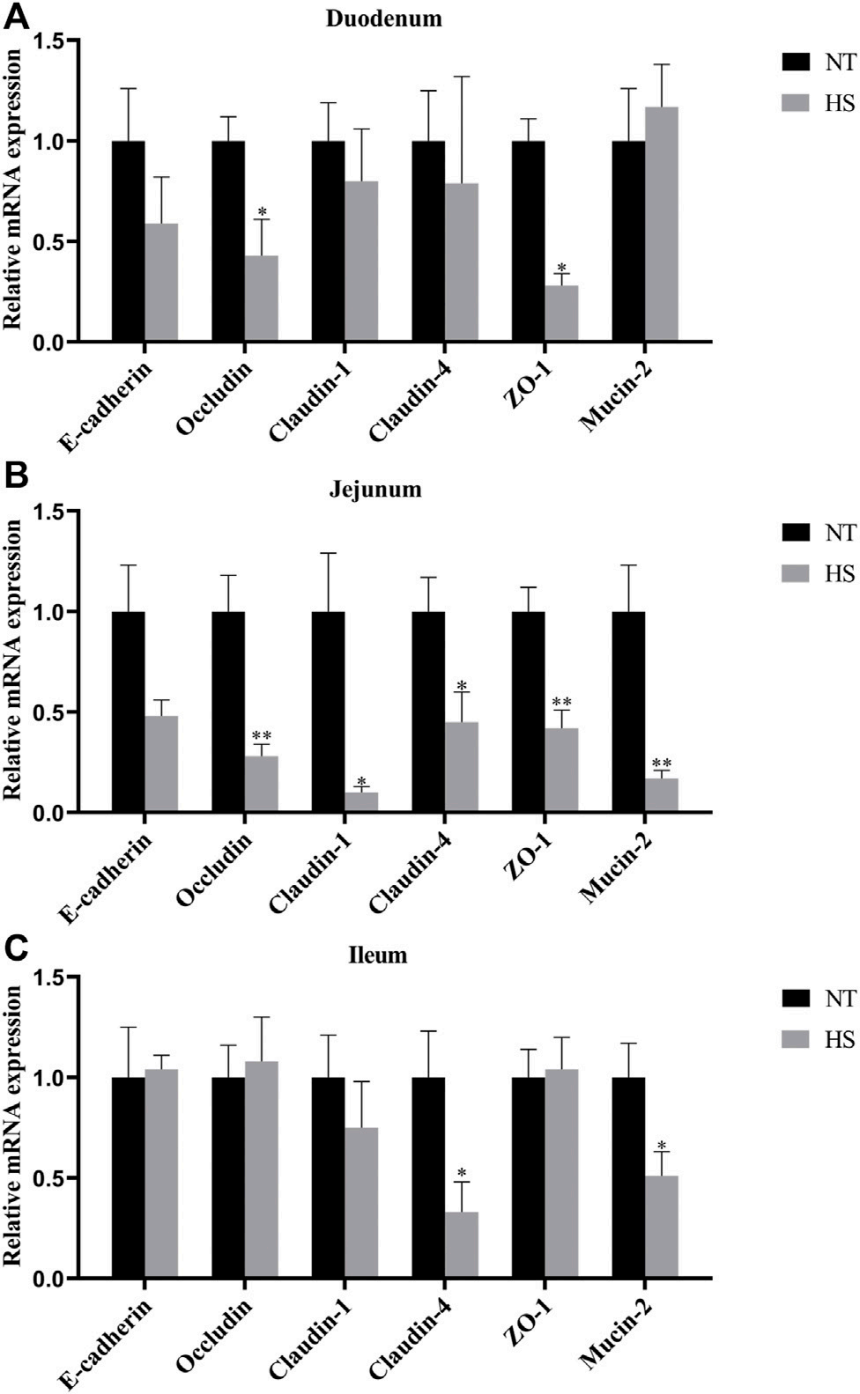
Effects of heat stress on relative mRNA expression of barrier function-related genes of duodenum **(A)**, jejunum **(B)** and ileum **(C)** in indigenous broilers (n = 5/group). NT, normal treatment group (21.3 ± 1.2°C, 24 h/day), HS, heat stress group (32.5 ± 1.4°C, 8 h/day). *p < 0.05, **p < 0.01.

The relative mRNA expression levels of intestinal barrier function-related genes are illustrated in Figure 3. HS down-regulated the mRNA expression of *Occludin* and *ZO-1* in duodenal mucosa (*p* < 0.05). The mRNA expressions of *Occludin, Claudin-1, Claudin-4, ZO-1* and *Mucin-2* in jejunal mucosa were down-regulated (*p* < 0.05) by HS exposure. Meanwhile, the mRNA expressions of *Claudin-4* and *Mucin-2* in ileal mucosa were down-regulated (*p* < 0.05) in broilers under HS.

### Cecal Microbial Community

**FIGURE 4:**
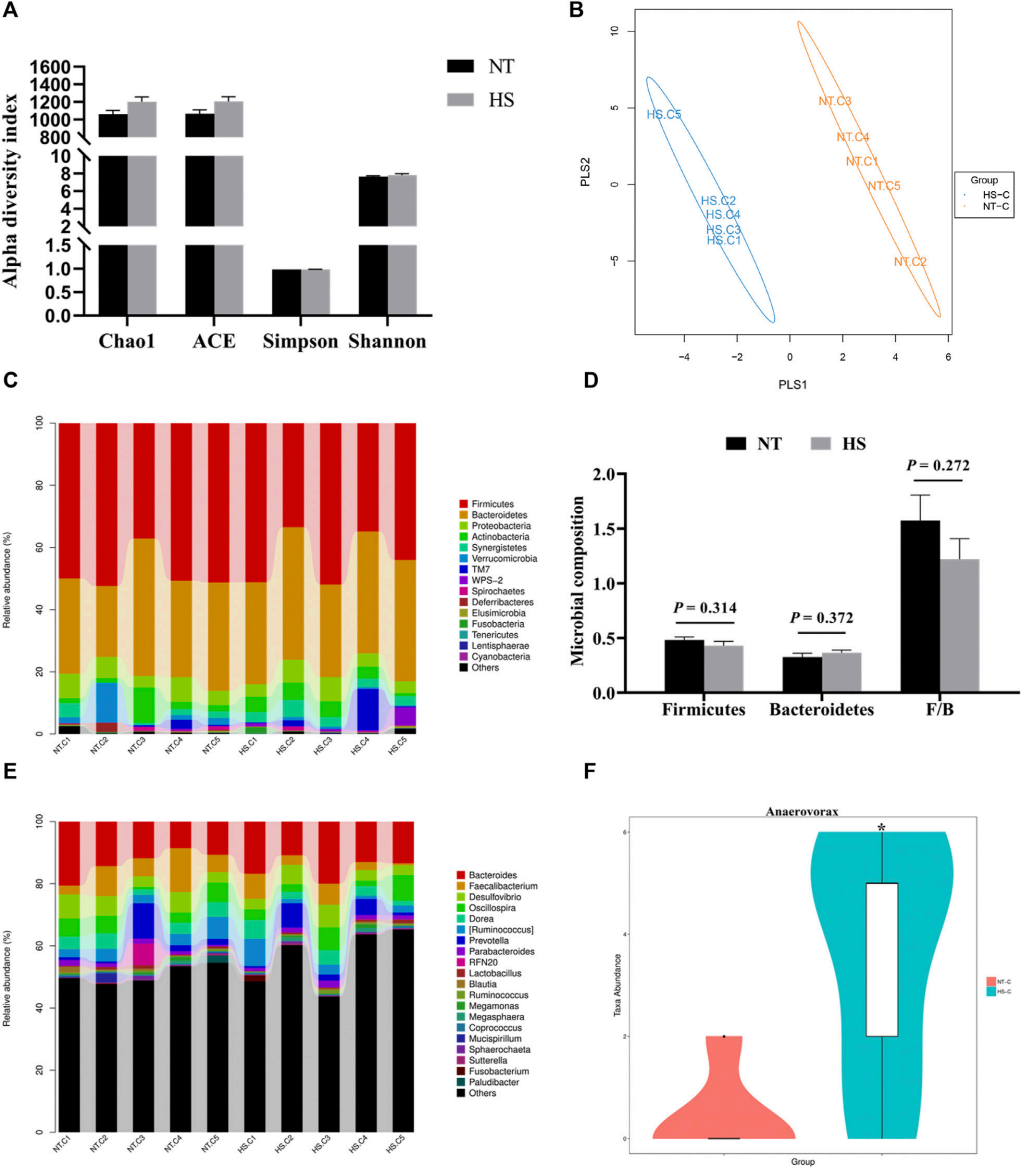
Effects of heat stress on cecal microbial community in indigenous broilers (n = 5/group). **(A)** alpha diversity; **(B)** beta diversity; **(C)** cecal microbial composition at the phylum level; **(D)** impacts of HS on the cecal relative abundance of *Firmicutes*, *Bacteroidetes* and the ratio of *Firmicutes/Bacteroidetes* (F/B) at the phylum level; **(E)** cecal microbial composition at the genus level; **(F)** impacts of HS on the cecal relative abundance of *Anaerovorax* at the genus level; NT, normal treatment group (21.3 ± 1.2°C, 24 h/day), HS, heat stress group (32.5 ± 1.4°C, 8 h/day). *p < 0.05.

There were no significant differences (*p* > 0.05) on alpha diversity indexes (Chao 1, ACE, Simpson, and Shannon index) between the NT and HS groups (Figure 4A). The beta diversity results showed that the consistency of intra-group samples in each treatment was good (Figure 4B). Regarding the relative abundance of cecal microbiota at the phylum level, HS exposure did not significantly affect the relative abundance of microbiota (Figure 4C, *p* > 0.05). Notably, the relative abundance of *Firmicutes* in HS broilers’ cecum was lower than NT group, and the relative abundance of cecal *Bacteroidetes* in broilers under HS was higher than NT group. Also, the ratio of *Firmicutes/Bacteroidetes* (F/B) was reduced by HS. However, none of these differences reached a significant level (Figure 4D, *p* > 0.05). At the genus level, it was found that HS exposure significantly increased the relative abundance of *Anaerovorax* in cecal digesta (Figures 4E,F, *p* < 0.05).

### Cecal Metabolome

**FIGURE 5:**
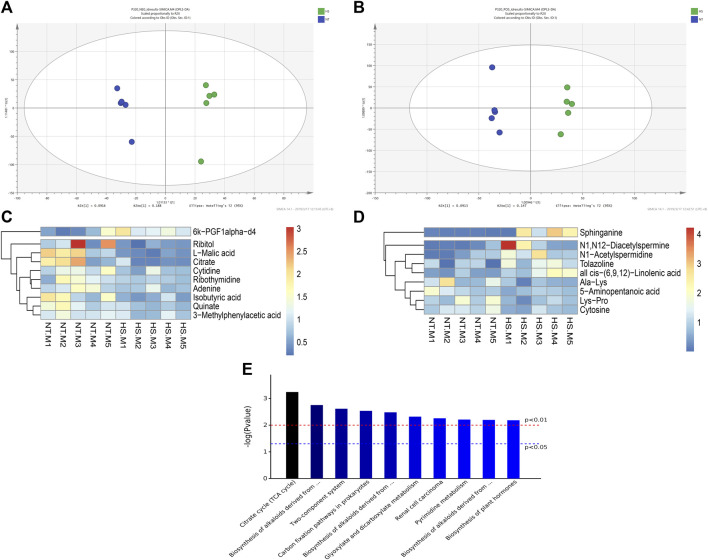
Effects of heat stress on cecal metabolome in indigenous broilers (n = 5/group). **(A)** Orthogonal partial least squares discriminant analysis (OPLS-DA) under negative ions mode (NEG); **(B)** OPLS-DA under positive ions mode (POS); **(C)** Cluster heatmap analysis of samples under positive ions mode (POS); **(D)** Cluster heatmap analysis of samples under negative ions mode (NEG); **(E)** Kyoto Encyclopedia of Genes and Genomes (KEGG) metabolic pathways enrichment analysis; NT, normal treatment group (21.3 ± 1.2°C, 24 h/day), HS, heat stress group (32.5 ± 1.4°C, 8 h/day).

The cecal metabolome results are presented in Table 5 and Figure 5. According to the screening criteria (*p* < 0.10, VIP>1), a total of 19 differential metabolites (containing negative and positive ions mode) were identified between the NT and HS groups. Among the metabolites, five metabolites were significantly down-regulated (*p* < 0.05) by HS exposure, including Cytosine, L-Malic acid, Citrate, Isobutyric acid, and Quinate. In addition, four metabolites were significantly up-regulated (*p* < 0.05) by HS exposure, including all cis-(6,9,12)-Linolenic acid, N1-Acetylspermidine, Sphinganine, and 6k-PGF1alpha-d4. The KEGG metabolic pathways analysis revealed that the differential metabolites mainly enriched (*p* < 0.01) in 10 signaling pathways, including the Citrate cycle (tricarboxylic acid, TCA cycle), biosynthesis of alkaloids derived from histidine and purine, two-component system, carbon fixation pathways in prokaryotes, biosynthesis of alkaloids derived from terpenoid and polyketide, glyoxylate and dicarboxylate metabolism, renal cell carcinoma, pyrimidine metabolism, biosynthesis of alkaloids derived from ornithine, lysine and nicotinic acid, and biosynthesis of plant hormones.

**TABLE 5:** Effects of heat stress on cecal differential metabolites in indigenous broilers.

Metabolites	Ratio (HS/NT)	*p*-Value	VIP
NEG			
Ala-Lys	0.451	0.095	1.895
Lys-Pro	0.567	0.093	1.917
Cytosine	0.633	**0.041**	2.164
5-Aminopentanoic acid	0.641	0.078	1.980
Tolazoline	2.145	0.060	1.918
all cis-(6,9,12)-Linolenic acid	2.806	**0.012**	2.471
N1-Acetylspermidine	3.064	**0.032**	2.214
N1, N12-Diacetylspermine	5.933	0.087	1.771
Sphinganine	10.863	**0.014**	2.577
POS			
L-Malic acid	0.262	**0.005**	2.761
Ribitol	0.285	0.064	2.166
Citrate	0.345	**0.045**	2.286
Isobutyric acid	0.473	**0.031**	2.326
Quinate	0.627	**0.027**	2.368
Cytidine	0.671	0.064	2.054
Ribothymidine	0.680	0.091	1.819
Adenine	0.705	0.084	1.827
3-Methylphenylacetic acid	0.762	0.071	2.063
6k-PGF1alpha-d4	1.990	**0.047**	2.001

NT, normal treatment group, the broilers reared at 21.3 ± 1.2 °C throughout the experimental period; HS, the broilers reared at 32.5 ± 1.4 °C for 8 h/day (9:00 a.m. to 17:00 p.m.); VIP, variable importance projection; NEG, negative ions mode; POS, positive ions mode; Bold values, *p* <0.05. There were 5 selected broilers per group for the determination of the cecal metabolome.

## Discussion

In practice, 32°C is the temperature that the indigenous broilers suffer from chronic HS in the environmentally equipped chicken house. Therefore, this study evaluated the changes in various parameters in indigenous broilers exposed to chronic HS at around 32°C for 4 weeks. Under the present thermal conditions, the decline of growth performance could be attributed to a series of adverse effects caused by HS, including electrolyte imbalance, endocrine disorders, energy-substance metabolism dysregulation, and organ injury (Chauhan et al., 2021). According to Lu et al. (2007), the HS exposure at 34°C for 3 weeks reduced the feed intake but had no significant impacts on BWG of slow-growing broilers (Beijing You chicken). Shao et al. (2018) demonstrated that cyclic HS (35°C for 7 days) decreased the BWG, feed intake, and feed/gain ratio of Chinese indigenous broilers (Xueshan broilers). In a study with Thai native chickens, HS exposure also reduced the BWG, but the HS conditions (35°C for 3 weeks) were not consistent with the current study (Malila et al., 2021). A recent meta-analysis study for fast-growing broilers revealed that the feed intake and feed conversion ratio were not significantly affected by 1–21 days of HS (Andretta et al., 2021). Therefore, it was widely accepted that HS results in unfavorable effects on growth performance, but the unequal duration and intensity of HS cause various consequences in growth indicators. The genetic background is also needed to be considered, especially for the indigenous broilers, which have different metabolic and growth rates, resulting in a variety of tolerance to HS (Wasti et al., 2020).

In addition, similar to the results of carcass composition in this study, Lu et al. (2007) demonstrated that the abdominal fat rate was increased in slow-growing Beijing You chickens under HS. Previous studies also reported that the breast and thigh muscle yield was decreased by 12–17% in slow-growing broilers subjected to HS conditions (Shao et al., 2018; Liu et al., 2019). However, these findings contradicted some other reports, which found that HS exposure had no significant effects on abdominal fat and muscle yield of broilers (Humam et al., 2019; Al-Sagan et al., 2020; Malila et al., 2021). The differences could be due to the broiler species and HS intensity. It has been suggested that the lipolysis and lipolytic enzymes activity and lipid metabolism-related pathways were suppressed under HS exposure, which subsequently resulted in abdominal fat deposition in broilers (Luo et al., 2018; Guo et al., 2021a). Furthermore, HS reduced the nutrients utilization and the retention of protein synthesis (Zuo et al., 2015) and caused muscle protein breakdown to an amino acid, thereby providing substrates to hepatic gluconeogenesis responsible for energy supply, and these changes were through the regulation of the insulin-like growth factor (IGF)/rapamycin signaling pathway (Ma et al., 2018; Ma et al., 2021). Therefore, molecules related to lipid metabolism and proteolysis may have different responses to variable HS intensity, and the expression of the molecules is also affected by genetic background, leading to the differences in carcass traits.

Regarding the meat quality, HS challenge increased the drip loss and reduced the muscle pH in the present study, which is in agreement with previous studies (Lu et al., 2017; Cheng et al., 2018; Wen et al., 2019; Awad et al., 2020). As it is well known, chronic HS induces hypoxia and increases anaerobic metabolism, leading to compensatory metabolic acidosis and a rise in muscle lactic acid concentration (Zaboli et al., 2019). Besides, it was observed that more pyruvate converted to lactic acid in HS broilers’ muscle (Gonzalez-Rivas et al., 2020). Therefore, the increased lactic acid content could reduce the pH of meat, and a lower post mortem pH denatures the muscle proteins, which compromises the water-holding capacity and causes an increase in drip loss (Mckee and Sams, 1998). Besides, the muscle mitochondrial dysfunction induced by HS could cause a decrease in the aerobic metabolism of fat and glucose and an increase in glycolysis, and the pH was reduced while the drip loss was increased accordingly (Lu et al., 2017).

Redox balance plays a critical role in maintaining physiological functions. It is widely accepted that HS exposure causes oxidative damage to the tissues and organs (Liu et al., 2021a). The antioxidant enzyme system, including GSH-Px, SOD, and CAT, could protect the body against oxidative damage under normal physiological conditions (Sies, 2015). When broilers were affected by HS at the initial stages, the antioxidant enzyme system was stimulated in response to HS. However, as the HS situation continued, the antioxidant enzyme system was dysregulated, thus leading to oxidative stress in broilers (Chauhan et al., 2021). The current findings further confirmed the theory of chronic HS-induced oxidative stress in broilers, HS exposure reduced the antioxidant enzymes activity and/or elevated MDA levels in serum, muscle, and small intestines. Similar results were also reported by previous studies (Lu et al., 2017; Cheng et al., 2018; Hu et al., 2020; Liu et al., 2021b).

Nowadays, researchers have paid great attention to the gut health of broilers, and growing evidence pointed out that gut health is the main target of HS (Wasti et al., 2020). Under HS conditions, broilers increase peripheral blood flow to accelerate heat dissipation, which in turn redistribute the blood and reduce the blood flow to the intestinal epithelium, thus resulting in gut ischemia-hypoxic injury (Rostagno, 2020). Meanwhile, oxidative stress induced by HS can directly destroy the intestinal epithelial barrier function of broilers (Mishra and Jha, 2019). The physical barrier in the gut is critical to intestinal health, and the tight junctions (TJs) are pivotal components of the physical barrier, which are responsible for regulating paracellular permeability and intestinal homeostasis (Teng et al., 2020). He et al. (2020) reported that the HS impaired small intestinal villus-crypt structure and decreased the expression of gut TJs-related genes in indigenous broilers. Liu et al. (2021c) demonstrated that the genes expression of TJs was down-regulated by HS in yellow-feathered chickens. Liu et al. (2021b) reported that HS exposure decreased the villus height and mRNA expression levels of TJs such as *Occludin* and *ZO-1* in the duodenum of local broilers breed. In agreement with previous studies, the present study found that HS-induced damage to intestinal morphology and TJs. The impairment of morphology and TJs could increase intestinal permeability, cause gut inflammation, reduce nutrients absorption, and consequently decrease BWG (Vandana et al., 2021).

The cecal microbial community was analyzed using 16S rRNA high-throughput sequencing; results showed that HS did not significantly impact the alpha diversity index. Consistently, Liu et al. (2021c) found that the alpha diversity of cecal microbiota was not affected by long-term HS exposure in yellow-feathered broilers. Wasti et al. (2021b) reported that HS had no significant effects on cecal microbial alpha diversity, such as Shannon entropy and Simpson’s index in HS Cobb 500 chicks. Prasai et al. (2016) also suggested that the chicken’s gut health was generally related to the changes in some specific microbiota rather than the dramatic alterations of the overall microbial-ecological diversity. Regarding the microbial composition, there were no significant differences in the relative abundance of microbiota at the phylum level. This is in accordance with the previous report by Shi et al. (2019), who observed that 28 days of HS did not significantly affect the cecal microbial composition at the phylum level in yellow-feathered broilers. Similar findings of fast-growing broilers were also reported in previous studies (Wang G. et al., 2021; Wasti et al., 2021b). It is worth noting that although there were no significant changes at the phylum level, the F/B ratio was reduced by HS. The ratio of F/B is an important marker of intestinal microbiota homeostasis (Lee and Hase, 2014), implying that HS affected the cecal microbiota balance. To delineate this effect, we further analyzed the microbial composition at the genus level and found that HS promoted the relative abundance of *Anaerovorax*. Similarly, the fecal *Anaerovorax* level was elevated after the toxins (tributyltin) challenge in rats, and the *Anaerovorax* could reduce the L-glutamic acid contents, thus impairing the intestinal barrier function (Yuan et al., 2020). Therefore, HS-induced injury of intestinal morphology and TJs may be associated with gut microbiome mediation in this study. However, the mechanism by which chronic HS influences intestinal microbiota is intricate and remains elusive. Thus, further works are needed to understand the role of gut dysbiosis in HS response and their correlation with the intestinal barrier function of broilers.

In this study, 19 differential cecal metabolites were identified in broilers after HS challenge based on metabolomics analysis. These mainly include organic acid, amines, and pyrimidine, and the KEGG analysis showed that these metabolites are enriched in TCA cycle, alkaloid synthesis, carbon fixation pathways, pyrimidine metabolism etc. The findings are similar to the study of serum metabolome in broilers under HS (Lu et al., 2018). In particular, the cecal contents of L-malic acid, citrate, and isobutyric acid were decreased by HS, and the L-malic acid was the most obvious of these changes. As an organic acid, L-malic acid has been reported to prevent ischemic injury (Tang et al., 2013), and the antimicrobial effect of L-malic acid was confirmed previously (Coban, 2020). It was found that the dietary L-malic acid displays positive effects on growth performance and gut health in quails (Ocak et al., 2009). Dietary citric acid also showed promotion effects on the gut health of broilers (Nourmohammadi and Afzali, 2013; Kammon et al., 2019). Isobutyric acid is a short-chain fatty acid and is beneficial for gut health (Ye et al., 2020). Accordingly, the impaired intestinal barrier may also be attributed to the reduced L-malic acid, citrate, and isobutyric acid levels in the cecum of broilers under HS. On the other hand, the L-malic acid and citric acid are involved in the TCA cycle (Goldberg et al., 2006). The results suggest that HS profoundly influenced the TCA cycle through metabolites, thus negatively affecting nutrient metabolism. Furthermore, HS reduced the cecal quinate content may deplete the gut antioxidant capacity as the quinic acid is a substrate for antioxidants (Pero et al., 2009). The HS-induced reduction of cytosine has deleterious consequences for nucleic acid synthesis, and an increase in metabolites, such as all cis-(6,9,12)-linolenic acid, N1-acetylspermidine, sphinganine, and 6k-PGF1alpha-d4, may be the response molecules to HS and which could be used as potential stress biomarkers (Lu et al., 2018).

**FIGURE 6:**
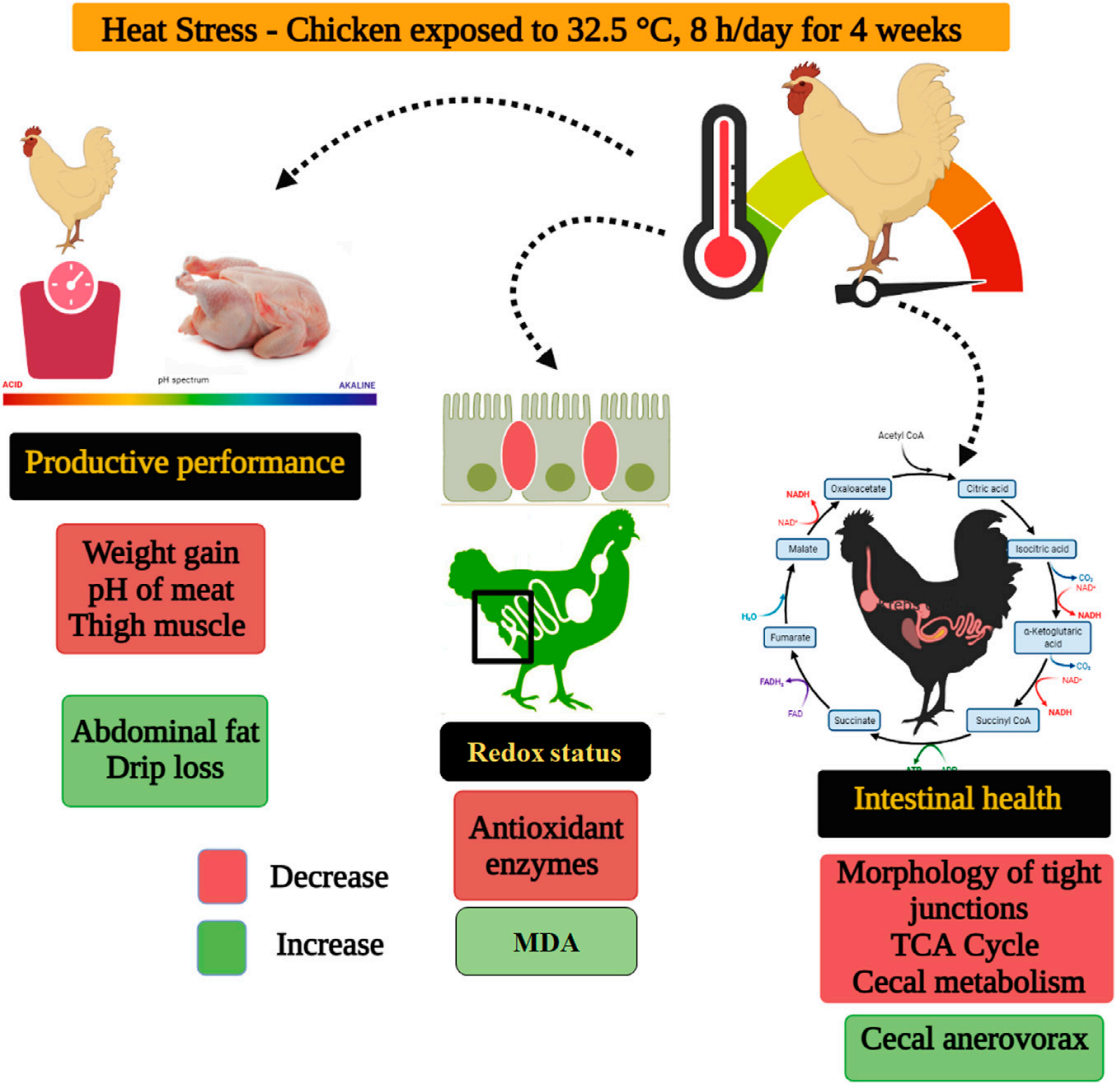
Proposed mechanism of heat stress on indigenous broilers (Huaixiang chickens).

Integrated analysis parameters that have been detected in this study (Figure 6), HS exposure negatively affected the production (weight gain, carcass and meat quality), redox status, and intestinal health (morphology, TJs, cecal microbiota and metabolome) of indigenous broilers. As oxidative stress causes organ dysfunction and tissue injury (Liu et al., 2021a), the reduced redox balance may be responsible for the deterioration of weight gain, meat quality, and gut health. Furthermore, the decline of weight gain may also be due to the impaired intestinal morphology and TJs function and the disturbances in cecal microbiota and metabolome. The novelty is that the cecal *Anaerovorax* and organic acid metabolites have the potential to be new regulatory targets for gut health in heat-stressed indigenous broilers, especially the L-malic acid. Whether the addition of exogenous L-malic acid alleviates HS-induced intestinal damage in slow-growing broilers, warrants further investigation.

## Conclusion

Chronic heat stress reduced the body weight gain and the antioxidant capacity, disrupted the intestinal physical barrier such as morphology and tight junctions, increased the relative abundance of *Anaerovorax* in the cecum, and lowered the metabolites related to gut health such as L-malic acid in indigenous broilers. These results could expand the understanding of the effects of heat stress on physiological changes and intestinal health in slow-growing broiler chickens, especially the cecal microbiome and metabolome findings.

## Data Availability

The data presented in the study are deposited in the NCBI repository, accession number: PRJNA816571.
